# Ecological view of carcinogenesis: Niche‐altering (NC^3^) mechanisms

**DOI:** 10.1002/cai2.54

**Published:** 2023-02-21

**Authors:** Mesut Tez

**Affiliations:** ^1^ Department of Surgery, Ankara City Hospital University of Health Sciences, Cankaya Ankara Turkey

**Keywords:** cancer, niche, niche altering mechanisms

Over the last 30 years, a new actor in the area of ecology and evolution has emerged: niche construction theory (NCT). The fundamental premise of NCT is that organisms may actively influence evolutionary processes by modifying their circumstances [1]⁠.

According to Trappes et al., [2] the concept of niche construction should only be applied to species that alter their environment, which they believe to be its intuitive scope. They add two more terms to describe the ways that organisms engage with their surroundings and so influence their niches: when organisms choose their environment, this is known as niche choice, and when they adapt their phenotype to fit their environment, this is known as niche conformance. These three concepts are often referred to as niche‐altering mechanisms, or NC^3^ mechanisms: niche conformance, niche construction, and niche choice. This is in line with definitions of mechanisms developed in scientific philosophy [2]⁠.

Cancer was attempted to be explained using NC^3^ mechanisms in this essay.

## 1 CANCER AS NICHE CONFORMANCE (INITIATION OF CANCER)

Carcinogenesis is a three‐stage process that includes initiation, progression, and metastasis [3]. Cancer initiation is an example of niche conformance. Phenotypic plasticity, or an organism's ability to develop distinct phenotypes in response to environmental variation, is involved in niche conformance. Unlike phenotypic plasticity, niche conformance also includes how phenotypic adjustment leads to changes in the phenotype‐environment match and fitness [2]⁠.

The constant replacement of old cells with newly formed healthy differentiated cells (progeny) originating from adult stem cells regulates epithelial tissue turnover. Each differentiated cell in an organism expresses a subset of all the genes found in that species' genome. The pattern of gene expression defines a differentiated cell type (hepatocyte, enterocyte, and so on). Adult stem cells are thought to reside in many tissues within a niche formed by a group of cells and an extracellular matrix that provides an optimal environment for the adult stem cell. Furthermore, adult stem cells are resistant to apoptosis, making them more resistant to the damaging effects of environmental stress than their progeny [4, 5]⁠⁠.

The stress stimulus exposure consists of all current environmental parameters (including the stress stimulus) as well as the organism's phenome (all features of the organism at that moment). The phenomenon of ‘this particular moment’ bears the effects of all previous encounters; the organism has plasticity (the phenomenal repertoire it can demonstrate in response to environmental changes) and abilities due to all previous processes (both from the organism's own life process and from the processes of its ancestors). The organism's plasticity in the face of a stress stimulus is related to its genome, epigenome, phenom, and environment, which it carries as a result of all previous experiences and evolutionary processes. After the industrial revolution, the majority of stressors (mutagen or non‐mutagen) were introduced into our modern lives (hostile‐chaotic environment). Our forefathers were never exposed to the chemical, biological, and physical agents that we are exposed to on a daily basis. Simultaneously, we are getting most of our calories from sugar and high‐fructose corn syrup and getting much less exercise. A hostile environment is one that is unpredictable. This environment can also be defined as one in which a suitable adaptation solution cannot be found. The lack of or delay in responding to a hostile environment also implies that detection by a sensor may result in a suboptimal response if the environment changes during the delay time. As a result, organisms have no choice but to respond to future uncertainty with the best answer they can provide. Response strategies that are deterministic‐stochastic are the best options. Such mixed deterministic‐stochastic strategies exist naturally. However, which stochastic strategy is best (chance/random, bet‐hedging, or chaos)? Random or bet‐hedging strategies are insufficient to explain adaptation to a hostile environment in a reasonable time. Previously‐unknown stress reduces the lifespan of differentiated cells and somatic stem cells, causing them to rapidly and reversibly switch to producing an excess of progeny to regenerate the lost tissue. A chaotic system is constantly producing new information that could not have been predicted at the outset. “Chaos is learning without a teacher,” to put it succinctly. A chaotic system is constantly producing new information that could not have been predicted at the outset. Furthermore, chaos allows the system to store information. Cancer can be initiated by new phenomes that emerge as a result of chaotic dynamics [6]⁠.

Niche conformance can be applied to irreversible developmental changes across an individual's lifespan (nonlabile traits) as well as reversible modifications in response to the present environment (labile traits).

## 2 CANCER AS NICHE CONSTRUCTION (PROGRESSION OF CANCER/TUMOR TISSUE)

Tissues are made up of cells and extracellular matrix (ECM) and groups of tissues come together in structural and functional units to create organs. The organism is formed through the interaction of several organs via blood and lymphatic vessels. Mechanisms of niche creation are distinguished by the focused individual, actively changing its surroundings. Tumor tissue is an example of niche construction. Solid tumors are not random collections of cells and ECM, but rather resemble organs, although architecturally and physiologically dysfunctional ones. They include many cell types and extracellular matrix components, and they grow through complicated interactions between these various tissue components, employing mechanisms that are typically similar to those utilized by developing organs. For example, the multilayered epithelium with weak polarity found in early breast tumors mimics the rapidly proliferating and invading epithelium of the developing mammary gland's terminal end buds. In both the developing and mature mammary glands, the transcription factor GATA3 supports epithelial differentiation, organization, and survival. It plays comparable roles in early breast cancer. However, when the carcinomas progress to later, less differentiated stages, GATA3‐negative, progenitor‐like cells are selected. Surprisingly, restoring GATA3 expression in late‐stage cancer cells results in the development of better differentiated and less metastatic tumors [7]⁠. Tumors, such as normal organs, interact with the rest of the organism. Thinking of tumors as niche construction mechanisms may help us better understand the processes that drive the development and progression of solid tumors.

## 3 CANCER AS NICHE CHOICE (METASTASES)

An individual relocating to a different habitat, known as habitat choice, is a paradigmatic kind of niche choice [2]*⁠⁠*. This is sometimes a transitory or context‐dependent option. Furthermore, competition among individuals for limited high‐quality habitat might result in phenotype‐environment correlations if certain individuals are driven into lower‐quality habitat (rather than choosing this based on their choice in the absence of competition). The finest illustration of cancer niche selection is liver metastasis. The liver is a major location for cancer metastasis, accounting for roughly 25% of all instances. Although metastases are widespread in noncirrhotic livers, many autopsy investigations have established that they are uncommon in cirrhotic livers. A review of the main ideas for the development of metastatic illness is adequate to explain the prevalence of liver metastases in the general population and the comparative rarity of metastases in the cirrhotic liver. The “seed and soil” hypothesis, proposed by Stephen Paget in 1889, posits that the “seed” of metastatic tumor cells would only become a full‐fledged metastasis if it reaches the suitable “soil” of a friendly environment, such as the otherwise healthy liver [8]⁠. The “seed and soil” hypothesis is a niche choice in terms of NC^3^ mechanisms.

## 4 EPILOQUE

In medical research, reductionism and specialization have led to key findings on both the processes of basic biological systems and the applications of how these systems might be managed. The reductionist approach has a blind hole in understanding and controlling complex biological systems such as cancer. The cross‐pollination of scientific disciplines and ideas from one field of study to another has resulted in new paradigms and significant transformations that can be described as unexplainable leaps of logic. There are strong parallels between complex ecological systems and cancer. For example, given ecologists' success in understanding eco‐evolutionary processes and managing pests under the integrated pest management framework, the question arises: might improved outcomes in cancer therapies be attained if oncologists begin to think like ecologists? NC^3^ mechanisms can be a good tool for this purpose.

## AUTHOR CONTRIBUTIONS

**Mesut Tez**: Writing – review & editing.

## CONFLICT OF INTEREST STATEMENT

The author declares no conflict of interest.

## ETHICS STATEMENT

Not applicable.

## INFORMED CONSENT

Not applicable.

## Data Availability

No data are available.
